# Avian Bornaviruses Escape Recognition by the Innate Immune System

**DOI:** 10.3390/v2040927

**Published:** 2010-04-01

**Authors:** Antje Reuter, Andreas Ackermann, Sonja Kothlow, Monika Rinder, Bernd Kaspers, Peter Staeheli

**Affiliations:** 1 Department of Virology, University of Freiburg, Freiburg, Germany; 2 Institute for Animal Physiology, University of Munich, Munich, Germany; 3 Clinic for Birds, Reptiles, Amphibians and Pet Fish, University of Munich, Oberschleissheim, Germany

**Keywords:** Borna virus, avian bornavirus, interferon

## Abstract

Like other pathogens that readily persist in animal hosts, members of the *Bornaviridae* family have evolved effective mechanisms to evade the innate immune response. The prototype of this virus family, Borna disease virus employs an unusual replication strategy that removes the triphosphates from the 5′ termini of the viral RNA genome. This strategy allows the virus to avoid activation of RIG-I and other innate immune response receptors in infected cells. Here we determined whether the newly discovered avian bornaviruses (ABV) might use a similar strategy to evade the interferon response. We found that *de novo* infection of QM7 and CEC32 quail cells with two different ABV strains was efficiently inhibited by exogenous chicken IFN-α. IFN-α also reduced the viral load in QM7 and CEC32 cells persistently infected with both ABV strains, suggesting that ABV is highly sensitive to type I IFN. Although quail cells persistently infected with ABV contained high levels of viral RNA, the supernatants of infected cultures did not contain detectable levels of biologically active type I IFN. RNA from cells infected with ABV failed to induce IFN-β synthesis if transfected into human cells. Furthermore, genomic RNA of ABV was susceptible to 5′-monophosphate-specific RNase, suggesting that it lacks 5′-triphospates like BDV. These results indicate that bornaviruses of mammals and birds use similar strategies to evade the host immune response.

## 1. Introduction

All successful persisting viruses must possess some efficient means to evade the immune system of the host. Infections with Borna disease virus (BDV) result in life-long viral persistence in cells of the central nervous system. Hallmarks of BDV persistence are high viral activity and abundant presence of viral antigens in infected cells [1]. These conditions are expected to create a milieu that triggers vigorous host cell responses if not actively counteracted by the pathogen. Other persisting RNA viruses such as hepatitis C virus face similar difficulties during replication in liver cells which they solve with the help of virus-encoding factors that greatly limit the synthesis of type I interferon (IFN) (reviewed in [2,3]). Interestingly, recent studies [4] indicate that BDV may employ a remarkably different strategy for evasion of the antiviral response of the host.

BDV is highly sensitive to type I IFN in cell culture. Type I was shown to efficiently protect Vero monkey cells and primary rat fibroblasts from *de novo* infection with BDV [1]. It was further shown to lower viral antigen levels in persistently infected cells [1]. In support of the view that IFN can restrict replication of BDV, mouse-adapted BDV was reported to multiply in cultured embryo cells derived from type I IFN receptor-deficient but not wild-type mice [5]. Surprisingly, however, *in vivo* virulence of BDV was not influenced by type I IFN in mice as no substantial differences were noted in the kinetics of virus spread in brains of wild-type and type I IFN receptor-deficient mice [5]. At least two different interpretations of these seemingly conflicting results are possible. First, the majority of BDV-susceptible cells in the brain may fail to respond to type I IFN. Second, BDV may suppress the synthesis of substantial amounts of type I IFN in infected brains. To distinguish between these two possibilities, infection studies were performed with transgenic mice that constitutively express an IFN-α gene in astrocytes. These experiments showed that IFN-α-transgenic mice were highly resistant to BDV infection [5], strongly arguing in favour of the second possibility.

How does BDV suppress IFN synthesis? Based on the findings discussed above, BDV could either actively block IFN induction pathways of the host or else hide from detection by receptors of the innate immune system. Evidence for both scenarios is available. Unterstab and coworkers [6] found that the P protein of BDV can inhibit the activity of TBK1, the principal regulatory kinase that activates IRF3 which is required for RIG-I-dependent induction of IFN genes in virus-infected cells. These results suggested that BDV-P may act as a decoy target for TBK1. However, this hypothesis could not satisfactorily explain why uninfected and persistently infected cells synthesized comparable amounts of IFN after infection with other viruses. An alternative scenario was suggested by Habjan and coworkers [4] who found that genomic RNA purified from BDV particles failed to stimulate RIG-I-dependent IFN synthesis in indicator cells, whereas purified genomic RNA of most other RNA viruses readily induced IFN gene expression. Additional studies showed that unlike in the case of viruses which are potent inducers of IFN, the 5′ termini of genomic RNA from BDV are not triphosphorylated and thus cannot be recognized by RIG-I. Since it was previously demonstrated that BDV employs an unusual replication strategy which includes a step that results in the specific 5′ trimming of viral RNA [7,8], these results suggested that BDV has developed this peculiar replication strategy in order to facilitate evasion of the host immune response [4].

Novel BDV-related viruses of birds, designated avian bornaviruses (ABV), were recently discovered [9,10] that cause proventricular dilatation disease in parrots [9–13] and non-psittacine birds [14]. Immunohistochemical and molecular analysis of organs from diseased birds suggested that ABV can replicate in a vast range of different organs and cell types of parrots [11,13,14]. ABV isolates are genetically more diverse than classical BDV and at least five genotypes have been identified to date [9,10,14]. Prototype members of genotypes 2 and 4 were successfully isolated from clinical specimens and shown to replicate in QM-7 and CEC32 quail cell lines [11]. However, no information regarding how ABV might cope with the IFN system of avian host cells has to date been available.

## 2. Results and Discussion

### 2.1. Sensitive monitoring of ABV infection using antiserum specific for the viral N protein

**Figure 1.**
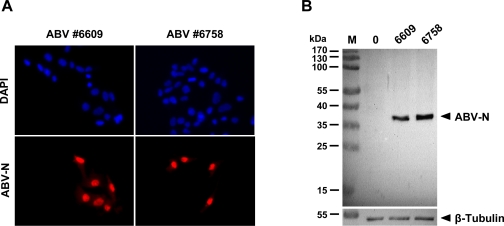
Rabbit antiserum specifically detecting the N protein of ABV in infected cells. **(A)** Indirect immunofluorescence analysis of QM-7 cells infected with ABV strains #6758 or #6609. Persistently infected and uninfected QM-7 cells were mixed at an initial ratio of 1:20 before plating. After overnight growth, the cultures were stained with antiserum (red) to visualize infected cells. DAPI (blue) was used for counter-staining. **(B)** Western blot analysis of lysates derived from QM-7 cells persistently infected with ABV strains #6758 or #6609. Uninfected QM-7 cells (0) served as controls. Pre-stained marker proteins (M) served as size standards.

We previously employed cross-reactive antisera originally made against the N and P proteins of BDV to detect the corresponding viral products in ABV-infected quail cells [11]. To detect ABV infections with greater sensitivity, we now generated a new rabbit antiserum that is directed against purified, histidine-tagged ABV-N protein (for details see Materials and Methods). When used for indirect immunofluorescence analysis of QM-7 quail cells, this new antiserum specifically recognized single cells infected with either ABV isolate #6758 (genotype 4) or isolate #6609 (genotype 2) in cultures of mainly uninfected cells (Figure 1A). Western blot analysis of lysates from QM-7 cells infected with the two different ABV isolates further showed that the new antiserum specifically detected a single viral protein of slightly less than 40-kDa, the predicted size of the translation product of the ABV N gene (Figure 1B). Taken together these results demonstrated that the newly generated rabbit antiserum represents an invaluable tool for a specific and sensitive detection of ABV-infected cells.

### 2.2. Type I IFN prevents efficient spread of ABV to uninfected quail cells

**Figure 2.**
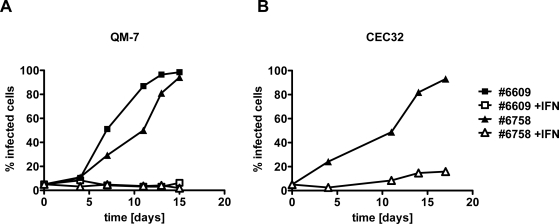
Effect of chicken IFN-α on the *de novo* infection of QM-7 **(A)** and CEC32 **(B)** quail cells with ABV strains #6758 and #6609. Virus exposure of uninfected cultures was started by mixing persistently infected and uninfected cells at an initial ratio of 1:20. The mixed cultures were then plated into two separate dishes which were subsequently kept in either plain medium (closed symbols) or medium containing 100 units per ml of chicken IFN-α (open symbols). The kinetics of virus spread was measured by visualizing ABV-N-positive cells in culture samples using indirect immunofluorescence analysis and by calculating the percentage of virus-infected cells.

To determine if type I IFN would influence the spread of ABV in quail cell cultures, we mixed persistently infected and uninfected cells at a ratio of approximately 1:20 before plating in two separate dishes. One of these cultures was then maintained in standard growth medium, whereas the second culture was maintained in growth medium supplemented with 100 units per ml of chicken IFN-α, which is highly cross-reactive on quail cells [15]. Each time when the cultures were split, a sample was used to determine the proportion of ABV-infected cells in the populations. If no IFN was present in the culture medium, the percentage of QM-7 cells containing easily detectable levels of N protein increased steadily and reached nearly 100% after approximately two weeks (Figure 2A). By contrast, if IFN was present in the culture medium, the percentage of ABV-N-positive cells remained below 10% during the entire observation period of 15 days (Figure 2A). The growth kinetics of ABV strains #6758 and #6609 under these two different culture conditions were not substantially different. A similar picture emerged when the protective effect of IFN against ABV strain #6758 was determined in quail CEC32 rather than QM-7 cells (Figure 2B). As in QM-7 cells, exogenously applied IFN inhibited the spread of ABV in cultures of CEC32 cells, indicating that ABV is highly sensitive to type I IFN.

### 2.3. Type I IFN inhibits the activity of ABV in persistently infected quail cells

**Figure 3.**
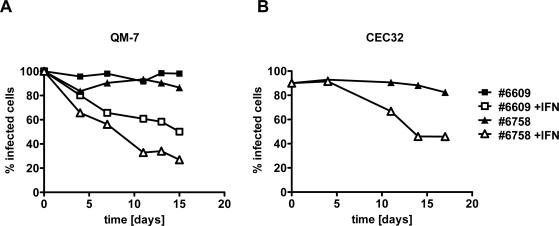
Effect of chicken IFN-α on quail cells persistently infected with ABV strains #6758 or #6609. Persistently infected QM-7 **(A)** or CEC32 **(B)** cells were kept in culture medium containing 100 units per ml of chicken IFN-α (open symbols) or plain medium (closed symbols). Virus inhibition over time was measured by visualizing ABV-N-positive cells in culture samples using indirect immunofluorescence analysis and by calculating the percentage of virus-infected cells.

We next investigated if type I IFN would also be able to reduce virus activity in persistently infected cells. We used persistently infected quail cell cultures in which >85% of individual cells contained high levels of ABV-N. At the beginning of the experiment, the cultures were plated into two dishes that either contained standard growth medium or growth medium supplemented with 100 units per ml of chicken IFN-α. As in the previous experiment, samples were analyzed for the presence of ABV-N by indirect immunofluorescence when the cultures were split into new dishes. Cultures of QM-7 cells persistently infected with ABV strains #6758 or #6609 grown in the absence of IFN contained at least 80% ABV-N-positive cells at each time point of analysis (Figure 3A). However, if the cultures were grown in IFN-containing medium, the percentage of ABV-N-positive cells declined steadily and reached values of about 50% or less after about two weeks (Figure 3A). Similar results were obtained if the antiviral activity of type I IFN was measured on CEC32 cells infected with ABV strain #6758 (Figure 3B). Thus, type I IFN seems to block a post-entry step of the ABV replication cycle.

### 2.4. Inefficient activation of type I IFN genes in ABV-infected quail cells

**Figure 4.**
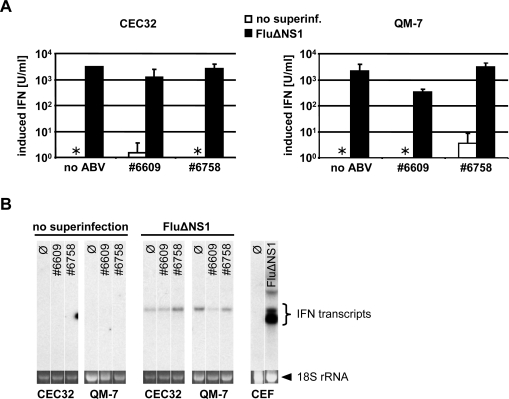
Synthesis of type I IFN by ABV-infected quail cells. **(A)** Antiviral activity in culture supernatants. IFN induction in uninfected cells and in cells persistently infected with ABV (strains #6758 and #6609, respectively) were compared (open bars). If the uninfected or ABV-infected cell cultures were super-infected with influenza mutant virus SC35MΔNS1 for 16 hours (filled bars), IFN was abundantly produced irrespective of whether or not the cells were persistently infected with ABV. The combined data from four independent experiments are shown. Asterisks indicate samples with IFN titers below the detection limit. **(B)** IFN-α transcript levels in the same cultures. After harvesting the supernatants, RNA was extracted from the various cultures and IFN-α transcript levels were determined by Northern blot analysis. RNA from uninfected (∅) or SC35MΔNS1-infected chicken embryo cells (CEFs) served as positive controls.

Since both ABV strains grew well in non-manipulated quail cells but only very poorly in cells that were treated with exogenous type I IFN, it appeared likely that ABV is a poor IFN inducer. To directly investigate this possibility, we measured ABV-induced expression of type I IFN genes by two independent methods. We first employed a sensitive bioassay [15] to determine if biologically active IFN was secreted by ABV-infected quail cells. These experiments showed that the supernatants of ABV-infected CEC32 and QM-7 cells contained extremely low levels of type I IFN that were not substantially higher than the background activity observed in uninfected cultures. Control experiments in which the influenza virus mutant strain SC35MΔNS1 (which is devoid of the IFN-antagonistic factor NS1) was used for IFN induction clearly showed that these cells are capable of synthesizing large amounts of type I IFN in response to virus infection (Figure 4A). The view that ABV is an extremely poor inducer of type I IFN was confirmed by direct IFN-α gene expression analyses by Northern blotting (Figure 4B). IFN-α mRNA was readily detectable in CEC32 or QM-7 cells infected with the positive control virus but not in cells infected with ABV strains #6758 and #6609 (Figure 4B).

### 2.5. Persisting ABV infection does not prevent IFN induction by super-infecting influenza virus

If ABV encoded a protein that actively inhibits type I IFN induction in infected cells, the response to super-infecting viruses should be strongly impaired in ABV-infected cells. To examine this possibility we compared uninfected and ABV-infected CEC32 and QM-7 cells with regard to their abilities to synthesize type I IFN in response to influenza virus infections. Cultures of ABV-free cells infected with SC35MΔNS1 contained about 2,000 units per ml of biologically active IFN (Figure 4A). QM-7 cells persistently infected with either ABV strains #6758 or #6609 responded comparably well to super-infection with SC35MΔNS1 (Figure 4A). Similarly, no major differences between ABV-infected and uninfected QM-7 cells were noted if Northern blotting was employed to detect IFN-α gene expression in response to SC35MΔNS1 infection (Figure 4B). Thus, collectively, these results strongly suggested that ABV does not employ an evasion strategy which relies on suppression of IFN synthesis by a virus-encoded antagonist.

### 2.6. No evidence for triphosphorylated 5′-termini of ABV genomic RNA

**Figure 5.**
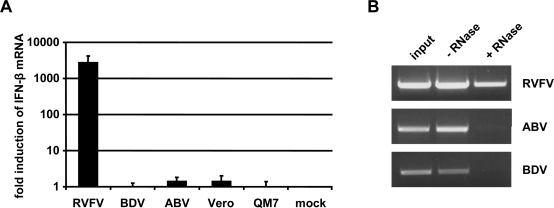
Evasion of host defence by ABV attributed to 5′-monophosphorylated genome ends. (**A**) 293T cells were transfected with total RNA isolated from uninfected Vero or QM-7 cells, or from cells infected with RVFV, BDV or ABV, respectively. At 8 h post transfection, RNA of the transfected cells was isolated and analyzed for IFN-β mRNA induction by qRT-PCR. Values are expressed relative to the mock control. Data shown reflect the average of two independent experiments with duplicates. (**B**) RNA isolated from partially purified virus preparations was digested at 37° for 30 min with the 5′-monophosphate-specific exonuclease Terminator™ that specifically digests 5′-monophosphorylated but not 5′-triphosphorylated RNA (+ RNase). An untreated sample (− RNase) served as control. Terminator™-resistant RNAs in samples from stocks of RVFV, BDV or ABV were visualized by RT-PCR analysis using primer pairs specific for the three viruses.

We explored the possibility that ABV and BDV might employ similar hiding strategies to escape detection by innate immune receptors. If genomic RNA of ABV lacked terminal 5′-triphosphates as recently reported for BDV [4], we would predict that RNA from ABV-infected cells fails to trigger an IFN response when transfected into suitable host cells. To examine this, total RNA from QM-7 cells persistently infected with ABV strain #6758 was prepared and transfected into human 293 T cells. At 8 h post infection, the transfected cells were lysed, RNA was prepared and IFN-β gene induction was assessed by quantitative RT-PCR (qRT-PCR). Total RNA from Vero cells infected with either Rift valley fever virus (RVFV) or BDV served as positive and negative controls, respectively. We found that RNA derived from both ABV- and BDV-infected cells failed to induce substantial levels of IFN-β mRNA in 293 T cells, whereas RNA from RVFV-infected cells induced the IFN-β gene very strongly (Figure 5A).

To determine if genomic RNA extracted from ABV particles lacks terminal 5′-triphosphates like BDV, we exposed purified RNA to Terminator™ exonuclease which specifically degrades 5′-monophosphorylated RNA and subsequently determined the efficacy of digestion by RT-PCR analysis using primer pairs that specifically amplify fragments of the different viral genomes. We observed that RNA prepared from ABV or BDV stocks exhibited a high degree of susceptibility to Terminator™, whereas RNA prepared from RVFV stocks exhibited a high degree of resistance to this exonuclease (Figure 5B). These results are compatible with the view that genomic RNA from ABV carries no 5′-triphosphates which may explain why ABV can infect host cells without triggering a strong IFN response.

## 3. Experimental Section

### 3.1. Viruses

The isolation of ABV strains #6609 and #6758 was previously described [11]. Influenza virus mutant SC35M-delNS1 was described previously [16]. RVFV clone 13 and BDV strain He/80_FR_ were described previously [4].

### 3.2. Production of antiserum against ABV-N

Histidine-tagged ABV-N protein was produced in *E. coli* using expression plasmid pQE9. Protein expression was induced with 0.03 mM Isopropyl-β-D-thiogalactopyranosid for 3 h after the bacterial culture reached the logarithmic growth phase at 28°C. Bacteria were lysed by sonification and the histidine-tagged ABV-N was purified using Ni-Agarose beads (Qiagen), dialysed against PBS for 48 h and used for immunization of a rabbit.

### 3.3. Cell lines and culture conditions

Quail cell lines QM-7 and CEC32 persistently infected with ABV strains #6758 or #6609 were available [11]. Both cell lines were cultured with DMEM supplemented with 8% fetal bovine serum, 2% chicken serum and antibiotics. For virus inhibition studies using chicken IFN-α, cells were seeded into 6-well dishes in medium supplemented with 100 units per ml of recombinant chicken IFN-α [17]. IFN treatment was continued for 15–18 days. Cultures were split as needed, usually every 3–4 days. Vero and HEK 293T cells were cultured with DMEM supplemented with 10% fetal bovine serum and antibiotics.

### 3.4. RNA isolation

RNA from cells or from purified virus was isolated with TriFast reagent (Peqlab) according to the manufacturer’s instructions. After addition of glycogen, RNA was precipitated by the addition of 1/10 volume of 3M sodium acetate and 2.5 volumes of ethanol.

### 3.5. Measuring IFN-β gene induction after RNA transfection

HEK 293T cells were transfected with RNA from infected or uninfected cells using Metafectene (Biontec) as described in the manufacturer’s protocol. Briefly, 2×10^5^ cells per well were seeded into 12-well tissue culture dishes some 18 h before transfection. RNA samples (1 μg) were diluted in 50 μl of OptiMEM (Gibco). In a separate tube, 3 μl of Metafectene was mixed with 50 μl of OptiMEM. Solutions containing RNA and Metafectene were then mixed and incubated for 15 min at room temperature before being pipetted onto the cells and incubated at 37 °C for 8 h.

RNA from the transfected HEK 293T cells was isolated using the NucleoSpin RNA II Kit (Macherey-Nagel) by following the manufacturer’s protocol. The RNA was eluted in 60 μl of RNase-free water, and the RNA concentration was determined. RNA samples (1 μg) were used for cDNA synthesis with QuantiTect reverse transcription Kit (Qiagen) according to the manufacturer’s instructions. IFN-β and γ-actin mRNA levels were analyzed in a LightCycler3 equipment using the QuantiTect SYBR Green PCR Kit (Qiagen) and QuantiTect primer sets: QT00996415 and QT00203763.

### 3.6. Digestion of RNA with 5′-monophosphate specific RNase

RNA from virus stocks containing RVFV clone 13, BDV strain He/80_FR_ or ABV strain #6758 was digested with Terminator™ (Epicentre) according to the manufacturer’s instructions. For each reaction, 0.5 μg of RNA was used. After 30 min incubation at 37 °C the reaction was stopped by precipitation of undigested RNA with ethanol. The pellet was re-dissolved in 11 μl of double-distilled water and used for cDNA synthesis with RevertAid H Minus First Strand cDNA Synthesis Kit (Fermentas) by following the manufacturer’s protocol. For quantification of viral products, 5 μl samples of the reverse transcription reactions were subjected to PCR analysis using primers 5′-gatcagatcaaattagtggc-3′ and 5′-ccaggaacaattaccttg-3′ for ABV, 5′-ttatcaagaagctagtgac-3′ and 5′-ctctgttccttgttgagg-3′ for BDV, and 5′-gacactcgagttaggctgctgtctttgtaagcctga-3′ and 5′-gacagacgtctcacatggacaactatcaagagctggcgat-3′ for RVFV.

### 3.7. Detection of ABV-infected cells by immunofluorescence analysis

To determine the percentage of ABV-positive cells in the various cultures, cell samples were seeded into 24-well dishes. After overnight growth, the cells were fixed with 3% paraformaldehyde and permeabilized with 0.5% Triton X-100. Rabbit antiserum to ABV-N was used typically at 0.1%. Bound antibodies were detected with Cy3-conjugated anti-rabbit IgG (Dianova). Cell nuclei were counter-stained with DAPI (Hoechst). For calculating the percentage of infected cells, at least 300 cells were counted.

### 3.8. Detection of ABV-N in QM-7 cell lysates by Western blotting

For testing the new antiserum by Western blotting, protein samples (10 μg) from infected and uninfected QM-7 cells were separated on a 12% SDS gel. After blotting on a PVDF membrane, the rabbit anti-serum at 1:500 dilution was applied for 90 min. Bound antibodies were visualized with peroxidase-conjugated anti-rabbit IgG (Dianova) and chemiluminescence chemisty (Pierce).

### 3.9. Bioassay for type I IFN

Type I IFN activity in cell culture supernatants was determined using a bioassay that employs quail CEC32 cells carrying an IFN-inducible firefly luciferase gene [15].

### 3.10. IFN induction using SC35M-delNS1

CEC32 or QM-7 cells were infected (multiplicity of infection of 1) with influenza A virus mutant (SC35MΔNS1) that lacks the IFN-antagonist factor NS1. After 1 h at room temperature, the virus inoculum was removed, fresh DMEM containing 2% fetal bovine serum was added and the cells were incubated at 37°C for 16 h.

### 3.11. Northern blot analysis

Cells were lysed with TriFast (Peqlab) and RNA was purified following the manufacturer’s protocol. After separation by electrophoresis and blotting on a nylon membrane, IFN transcripts were visualized by hybridization with a radioactively labeled chIFN-α1 cDNA probe.

## 4. Conclusions

Our results collectively indicate that the recently discovered avian bornaviruses evade the host IFN response by a strategy that resembles the strategy used by classical Borna disease virus, the prototype virus of the *Bornaviridae* family. Both ABV and BDV apparently have acquired mechanisms which allow them to hide very efficiently from recognition by innate immune receptors such as RIG-I. Presumably as a consequence of this successful hiding strategy which blocks IFN synthesis almost completely, neither ABV nor BDV seem to have developed additional mechanisms that would block the action of type I IFN in persistently infected cells.

Interestingly, all available evidence supports the view that bornaviruses do not encode factors which might actively repress virus-triggered signaling cascades that lead to the activation of type I IFN genes. The evasion strategies of bornaviruses thus differ markedly from strategies used by influenza, hepatitis C and other RNA viruses that rely on genes which code for accessory proteins that serve as IFN antagonists [2].

Circumstantial evidence suggests that the evasion strategy of the bornaviruses is surprisingly simple. These viruses trim the 5′ ends of their genomic and anti-genomic RNAs [8], thereby removing any tri-phosphorylated residues which could be sensed by innate immune receptors. It thus appears likely that the exceedingly complicated mode of genome replication of bornaviruses [7] has evolved under strong selection pressure from the host innate immune response.
